# Quantification of arthritic bone degradation by analysis of 3D micro-computed tomography data

**DOI:** 10.1038/srep44434

**Published:** 2017-03-14

**Authors:** Carl-Magnus Svensson, Bianca Hoffmann, Ingo M. Irmler, Maria Straßburger, Marc Thilo Figge, Hans Peter Saluz

**Affiliations:** 1 Applied Systems Biology, Leibniz Institute for Natural Product Research and Infection Biology - Hans-Knöll-Institute, Beutenbergstrasse 11a, 07745 Jena, Germany; 2 Cell and Molecular Biology, Leibniz Institute for Natural Product Research and Infection Biology - Hans-Knöll-Institute, Beutenbergstrasse 11a, 07745 Jena, Germany; 3 Friedrich Schiller University Jena, Germany; 4 Institute of Immunology, University Hospital Jena, Leutragraben 3, 07743 Jena, Germany; 5 Transfer Group Anti-infectives, Leibniz Institute for Natural Product Research and Infection Biology - Hans-Knöll-Institute, Beutenbergstrasse 11a, 07745 Jena, Germany.

## Abstract

The use of animal models of arthritis is a key component in the evaluation of therapeutic strategies against the human disease rheumatoid arthritis (RA). Here we present quantitative measurements of bone degradation characterised by the cortical bone profile using glucose-6-phosphate isomerase (G6PI) induced arthritis. We applied micro-computed tomography (*μ*CT) during three arthritis experiments and one control experiment to image the metatarsals of the hind paws and to investigate the effect of experimental arthritis on their cortical bone profile. For measurements of the cortical profile we automatically identified slices that are orthogonal to individual metatarsals, thereby making the measurements independent of animal placement in the scanner. We measured the average cortical thickness index (CTI) of the metatarsals, as well as the thickness changes along the metatarsal. In this study we introduced the cortical thickness gradient (CTG) as a new measure and we investigated how arthritis affects this measure. We found that in general both CTI and CTG are able to quantify arthritic progression, whilst CTG was found to be the more sensitive measure.

Rheumatoid arthritis (RA) is an autoimmune disease that affects approximately 1% of the adult population leading to pain, disability and, if not treated, significantly decreased life span^1^,^2^. Although RA is an exclusively human disease, animal models of arthritis provide invaluable tools to investigate the aetiology and to test therapeutic strategies. The degree of RA in a limb is routinely scored in a semi-quantitative way by the physician or the experimentalist, estimating bone degradation from X-ray images^3^. Although the scoring methods are clinically well established and tested, the score still depends on the individual’s judgement, thus resulting in a subjective evaluation of the disease progression. In the predominantly murine-based animal models of experimental arthritis, some concerns that hinder the direct studies of RA can be less serious. The most obvious ones are the caution due to patient radiation exposure, and the possibility of performing histology on the test subject in order to examine the arthritic progression directly.

In animal models, bone degradation caused by different types of arthritis is well documented^4^,^5^ but the severity is normally longitudinally judged by manual scoring, which is a semi-quantitative method. The *in vivo* application of computed tomography with micrometer resolution (*μ*CT) is an advancement in the “Three R’s” principle formulated by Russell and Burch in 1959^6^. The “Three R’s” are ethical guidelines for animal trials and correspond to: *refine, reduce* and *replace*. The use of longitudinal *in vivo* measurements will considerably reduce the number of necessary animals, whereas the longitudinal analysis can also refine the experiment itself by decreasing the suffering and distress experienced by the animals.

A standard approach to evaluate the effects of experimental arthritis on bones is to measure the bone mineral density (BMD) using *μ*CT^7^,^8^. BMD measurements require high radiation doses and the density needs to be concurrent with the scanning of a calibration phantom, which means that histological samples are used in most cases^7^,^9^. The utilisation of *μ*CT, positron emission tomography (PET) or magnetic resonance imaging (MRI) allows for longitudinal studies of the disease in single animals or in patients^10^,^11^, without the use of histological approaches applied to characterize BMD and malformations^5^,^12^. The use of *in vivo μ*CT in rodent models of arthritis has mainly focused on the major bones, *e.g*. femur or humerus, and certain models of osteoarthritis^13^,^14^,^15^. The studies that investigate the metatarsal/phalangeal area of mouse models focus on cartilage damage and try to approximate the bone density^16^,^17^.

It is known that the cortical structure is affected by different types of arthritis^18^,^19^,^20^,^21^,^22^ and in this paper we demonstrate that changes of both the average thickness and the longitudinal gradient of the thickness of the cortical bone are affected by experimental arthritis. We successfully overcame the technical difficulties related to obtaining this measure in a setting of *μ*CT scans of murine hind paws. We performed bone segmentation, followed by calculation of the cross section of the bones orthogonal to the centre line of the bone, and finally compacted the cortical bone profile into measurements that were useful for the discrimination between arthritic and healthy animals. Specifically, we examined the thickness and the gradient of the cortical bone in the metatarsals of mice, which is among the earliest-affected regions in animals immunised with glucose-6-phosphate isomerase (G6PI), inducing poly-arthritic disease^23^.

While the methods we used are applicable to other bone types, such as the tibia, here we focused on the metatarsals, because the changes in their cortical profile due to experimental arthritis have not been quantitatively examined before. The bones otherwise considered for cortical thickness analysis are mostly the tibia or the femur, as the isolation of these bones is much easier than of those in the crowded environment of the paws. For certain types of experimental arthritis, for example collagen induced arthritis, the knee joints show maximally increased bone metabolism in PET studies^24^. This is not the case in G6PI induced arthritis where the main increase in bone metabolism is localised to the distal joints, for example the metatarsophalangeal joints^5^,^25^. Another advantage of using the larger bones is that the trabecular compartments may also be analysed^18^,^22^. The loss of trabecular bone mass is another factor that is commonly shown to correlate with RA^19^,^22^, but for *in vivo μ*CT images of the metatarsals the resolution is not high enough to determine the trabecular structure. There has been progress in identifying single bones from CT and MRI of the carpus^26^,^27^,^28^,^29^, but these studies are not coupled with automatic determination of bone degradation. Here we developed a semi-automated method for the identification of metatarsals in the CT data of murine paws that are acquired non-invasively for a sensitive analysis of cortical bone degradation in distal joint regions.

## Theory and Implementation

### Texture based segmentation

**Figure 1:**
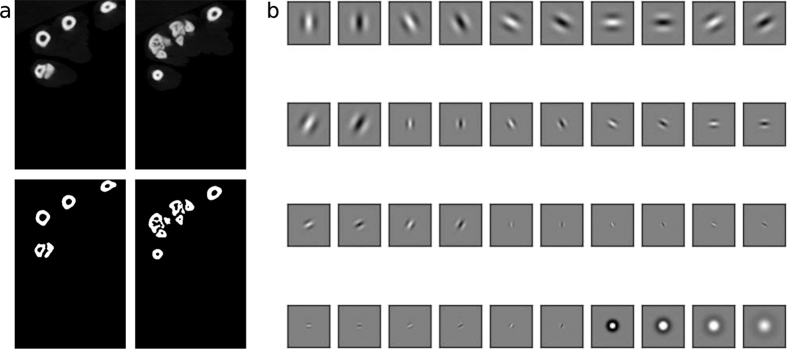
(**a**) Example of two CT slices (top row) segmented by the texture based approach (bottom row). The texture based approach was capable of identifying closely lying bones that are separated by tissue that is of higher intensity than the general background. (**b**) The filter bank used following Malik *et al*.^32^. The first 36 filters are Gabors with *ϕ* ∈ [0, *π*/2], *θ*_*m*_ ∈ [0, *π*/6, *π*/3, *π*/2, 2*π*/3, 5*π*/6], 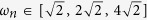 in all combinations. The last four are DoGs with paired standard deviations[*σ*_*m*_, *σ*_*n*_] ∈ {[0.075, 0.125], [0.125, 0.175], [0.175, 0.225], [0.225, 0.275]}.

Texture is normally defined as differences in an image that cannot be described by means of first and second order statistics (*i.e*. intensity and variance). The data set needed to be segmented to identify individual bones both in every image and across slices, in order to create the three dimensional paw. The data seen in Fig. 1a can superficially be judged to be simply segmented by intensity, for example by Otsu’s method^30^. In fact, thresholding of our data was problematic because individual bones can lie close to each other and thresholding was not able to separate these. In between the bones there were different types of tissue that also affected the image quality. Especially around joints, there was often cartilage, which means that the background inference was larger where the bones were in close proximity. Texture depends not only on the pixel value, but also on its immediate surroundings. Consequently, a pixel that was in between two lighter regions were less likely to be considered foreground than a similarly intense pixel surrounded by equally intense neighbours. The texture based segmentation, as described here, has no need for parameter tuning when applied to new data sets, which is in contrast to the dual threshold method developed by Buie *et al*.^18^ where the thresholds have to be adjusted for new data sets. The use of edge information incorporated in texture based segmentation was very good at segmenting individual bones and worked well in our case as represented in Fig. 1a. In Fig. S1 of the Supplementary Material we demonstrate the difference between the texture based segmentation and a thresholding and morphological opening scheme. In Fig. S2 of the Supplementary Material we quantitatively show that the parameter-free, texture based segmentation gave a better result than a thresholding based segmentation algorithm. As a measure of segmentation accuracy, we used the Dice coefficient^31^ between manually segmented bones and bones segmented by the algorithms.

Texture based segmentation was performed by applying a filter, *F(x, y*), to the image. Many filter banks are inspired by the receptive fields found in the visual system of mammals, including the human visual system, because these are powerful texture discriminators. In cats and primates the receptive fields in the primary visual cortex are best fitted with Gabor functions, whereas those in the lateral geniculate nucleus (LGN) are best described by difference-of-Gaussians (DoGs). For this study we used a standard bank of filters introduced by Malik *et al*.^32^, which includes both Gabors and DoGs. The general form of a Gabor filter is


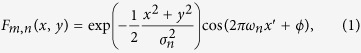



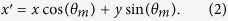


The Gabors are characterised by the frequency *ω*_*n*_ and the orientation *θ*_*m*_. In general, the window can have different sizes in the *x* and *y* direction but for this study we used *σ*_*n*_ = *σ*_*x*_ = *σ*_*y*_, *i.e*. we imposed a circular window. The use of the common subscript *n* for both frequency and window size is because those are inversely proportional. The phase of the sinusoid is determined by *ϕ*. The two-dimensional DoG filter is


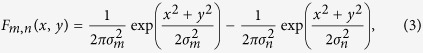


where *σ*_*m*_ < *σ*_*n*_. The realisation of these filters with different parameters following Malik *et al*.^32^ produced the filters in Fig. 1b. We used in total 36 Gabors spanning 3 spatial frequencies, 6 orientations and 2 phases. The phases were 0 and *π*/2 so that we had a quadrature pair of each filter^33^. Additionally we used 4 DoGs with a positive center, which gave us *N*_*F*_ = 40 filters in total. All the filters were convolved with each image *I(x, y*), according to


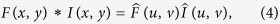


where 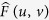 (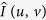) is the two-dimensional Fourier-transform of the filter (image). After convolution, the grayscale images, each of which had *M* × *N* pixels, became a dataset with *M* × *N* × *N*_*F*_ entries. Each pixel is represented by the response to each of the filters.

The filtered dataset, 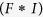 (*x, y*), was even more fractured than the original grayscale image and the task to segment the bone from background was still to be solved. This was accomplished by applying the *k*-means++ algorithm to all the pixels with *k* = 2. The *k*-means algorithm is a method for clustering data points into groups based on their distance to *k* cluster centres. The assumption here was that the filter responses of the background pixels are more similar to each other than to the responses of the bone pixels in a Euclidian sense. The specific advantage of *k*-means++ is that initial conditions are chosen with care so that the algorithm is faster and more accurate than implementations of *k*-means with uniformly randomly chosen initial conditions^34^. In Fig. 1a we show examples of how the texture based segmentation transformed the original data to a binary image. The *k*-means++ algorithm was implemented in the scikit-learn library^35^.

### Metatarsal Isolation

**Figure 2:**
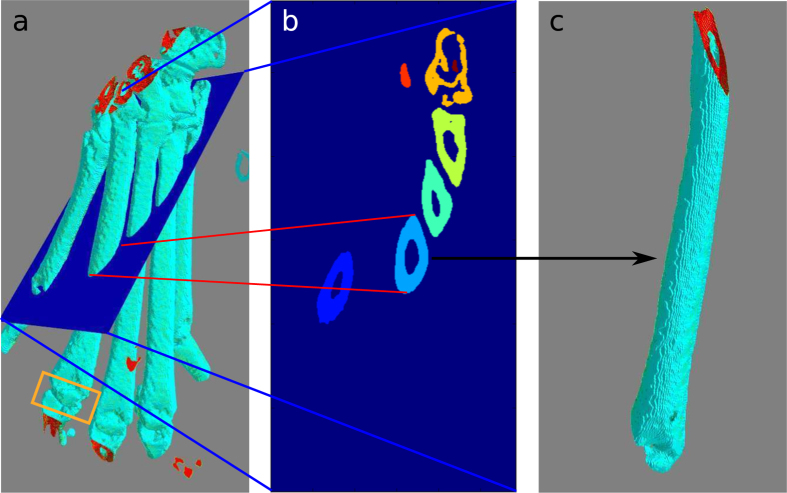
(**a**) The iso-surface of the segmented paw in 3D. The orange box marks the metatarsophalangeal joint of the number 4 metatarsal. (**b**) The slice corresponding to plane plotted in (**a**) with each bone labelled in a different colour. The light blue bone cross section represents the 4th metatarsal of the paw and this bone was tagged and then tracked across slices. The tracking stops when getting close to the phalange joint, the point for this was manually decided. (**c**) The isolated 4th metatarsal.

**Figure 3:**
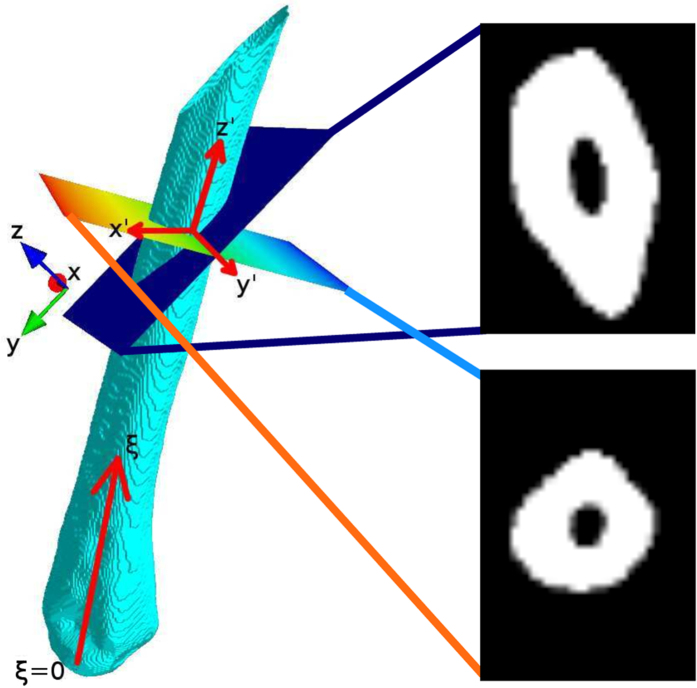
Overview of the identification of orthogonal planes. Three dimensional plotting was done using the Mayavi library^57^. The bone-centred coordinate system is denoted (*x*′, *y*′, *z*′). The dimension *ξ*, with *ξ* = 0 at the metatarsophalangeal joint, is bone specific and was used to measure how far up the metatarsal we are. On the metatarsal we have plotted the original scanning plane (dark blue) and the orthogonal plane (multicoloured) spanned by *x*′ and *y*′. To the right, we show the original slice with the projection of the bone on the original *x* − *y* plane (top) and the projection of the bone on the orthogonal *x*′ − *y*′ plane (bottom).

To measure the cortical bone thickness, we first needed to isolate the individual bones so that we could describe the surface using cylindrical coordinates with the bone centre set as the *z*-axis. The tracking started at a point where the metatarsals were easily identified manually and were then automatically identified in neighbouring slices. To isolate a metatarsal, for example the 4th metatarsal marked in Fig. 2a, we manually identified the desired bone somewhere between the joints, see Fig. 2b. The selected bone was identified in neighbouring slices based on the centre and the size of the objects by matching the regions with the smallest area difference that lie within the shadow of the region from the previous slice. In addition to the identification of the metatarsal of interest in Fig. 2b, the user was also required to provide the *z*-position of the metatarsophalangeal joint for the digit in question; this joint is marked by an orange box in Fig. 2a. These two interactions were the only two user inputs needed in our otherwise automated analysis. From each slice, the center of the metatarsal in question (*x, y, z*) was saved and this was used as a basis both for obtaining slices that are orthogonal to the bone and for calculating the cylindrical coordinates. This new axis running along the center of the bone is denoted *z*′ = *z*′ (*x, y, z*), as can be seen in Fig. 3, and is essential for the construction of orthogonal slices from where the cortical bone thickness was measured.

### Orthogonal Slices

The new bone-centred coordinate system was spanned by the orthonormal vectors **e**_*x*′_, **e**_*y*′_ and **e**_*z*′_, which was expressed in relation to the original base **e**_*x*_, **e**_*y*_ and **e**_*z*_. For each slice we translated the bone coordinate system such that its origin was lying in *O(i*) = [*x*_*i*_, *y*_*i*_, *i*], where *x*_*i*_ and *y*_*i*_ were the center of mass of respective bone in slice *i*. The relative positions of the original and bone-centred coordinate systems can be seen in Fig. 3. The normal to the orthogonal plane was in the direction **e**_*z*′_ and the other basis vectors were lying in the plane. The normal was defined by finding the local direction of the centreline at position *z* = *i* by the approximation


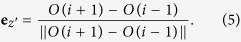


We expressed the base vector in the original coordinate system **e**_*z*′_ = *x*_*z*′_**e**_*x*_ + *y*_*z*′_**e**_*y*_ + *z*_*z*′_**e**_*z*_, where (*x*_*z*′_, *y*_*z*′_, *z*_*z*′_) were given by equation (5).The second basis vector in the plane was found by identifying the axis of rotation between **e**_*z*_ and **e**_*z*′_ which is given by


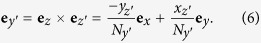


The normalising factor, 
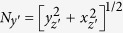
, ensured that we constructed an orthonormal basis. The final basis vector in the plane was given by the cross product between **e**_*y*′_ and **e**_*z*′_ which produced


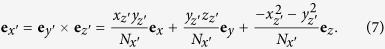


Here the normalisation factor was


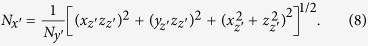


We could then move forward and backward between the original space, **e**_***x***_, and the bone-centred space **e**_***x***′_ through the transformations


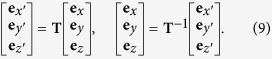


The transformation matrices were


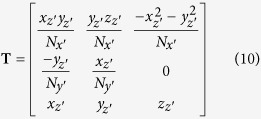


and


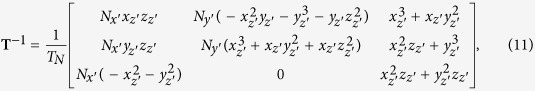


where 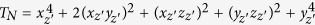.

With the transfer matrices between the two coordinate systems defined, we reconstructed slices that were locally orthogonal to each metatarsal. The bone thickness was measured directly from the orthogonal plane, independently of the angle between bone and scanning plane during the experiment. In Fig. 3, an example of orthogonal transformation of a metatarsal is shown.

### Cortical bone thickness measurements

**Figure 4:**
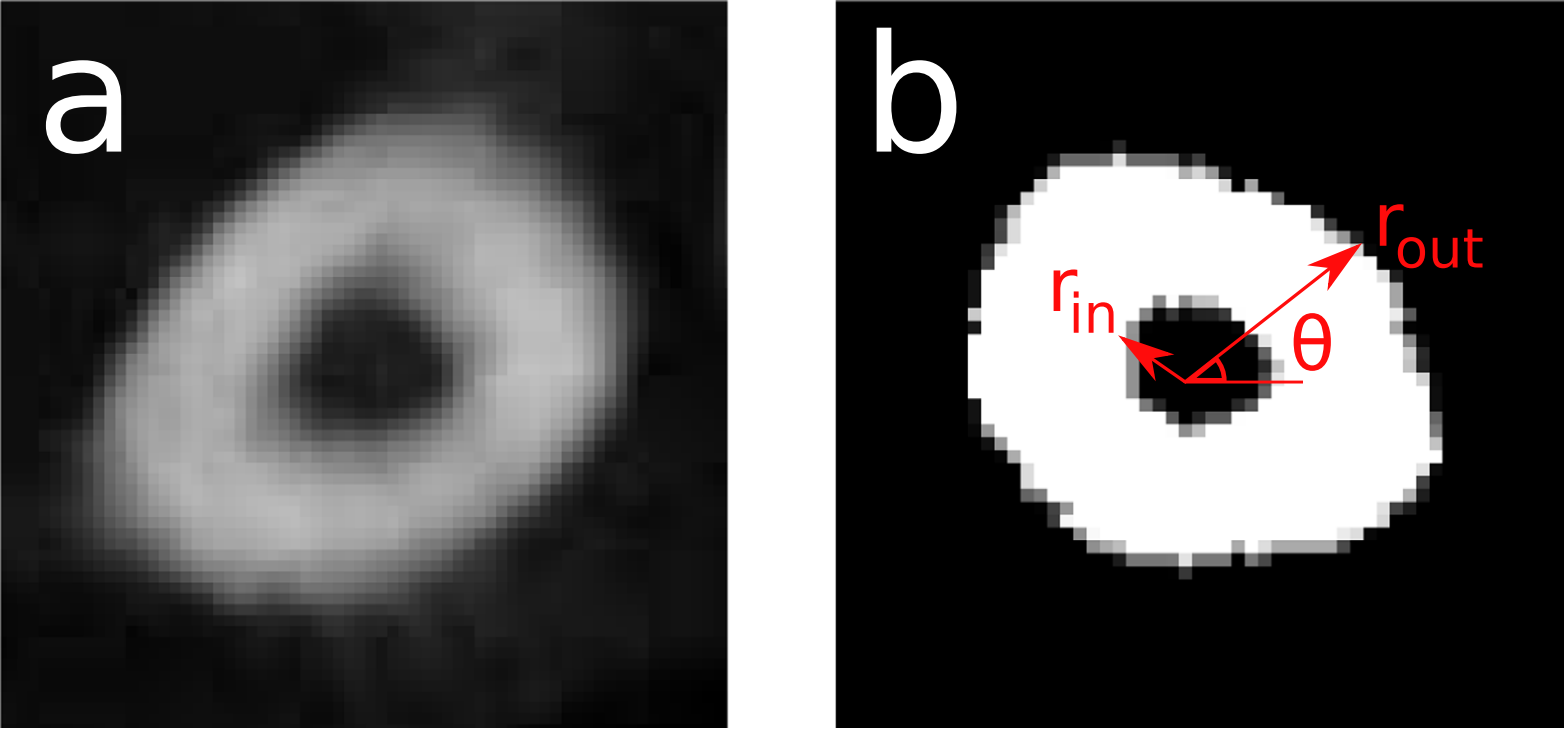
(**a**) Example of an original *μ*CT-slice at height *z*′ = *ξ*. (**b**) The orthogonal slice at height *z*′ = *ξ* with the outer and inner radii marked. These defined the thickness of the cortical bone, Ψ(*ξ, θ*), by taking the difference between the radii.

The cortical bone thickness was defined as the distance between the inner and outer perimeter, as seen in the segmented orthogonal slices. In the orthogonal slices we introduced cylindrical coordinates (*r, θ, z*′) where the origin was placed in the center of mass of the metatarsal for each slice, see placement *z*′ = *ξ* in Fig. 4a,b. The cortical thickness was defined as Ψ(*ξ, θ*) = *r*_*out*_(*ξ, θ*) − *r*_*in*_(*ξ, θ*), see Fig. 4b, where *ξ* was the longitudinal position on the metatarsal and *θ* was the angle. The radii of both perimeters were sampled at *N*_*θ*_ = 25 discrete *θ*’s where the sampling frequency was determined by the typical number of pixels of inner perimeters which ranged between 15–50 pixels. The number of *ξ*’s that were sampled was determined by the length of the metatarsal and the angle at which the metatarsal was scanned. To evaluate quantitative differences between Ψ(*ξ, θ*) for different experimental conditions, we first reduced the two-dimensional function to one dimension by taking the angular average


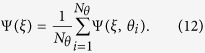


To this one-dimensional signal we fitted the parameters of a linear function


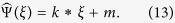


**Figure 5:**
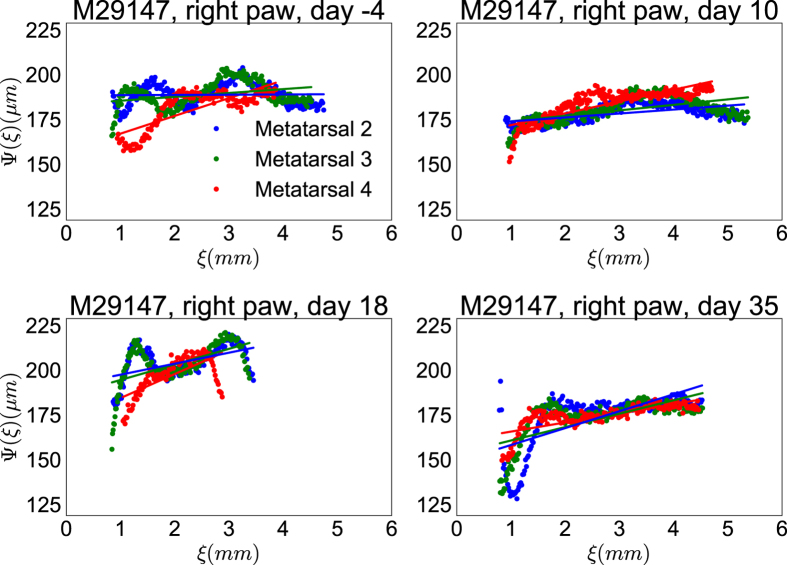
The evolution of cortical bone thickness, Ψ(*ξ*), along the three central metatarsals of the right hind paw for animal M29147. The animal was immunised at day 0 and scanned at days −4, 10, 18 and 35. The measured thickness of the cortical bones is shown with points in different colours, representing the different metatarsals, whereas the fitted function, 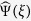, is shown as a solid line of the same colour as the metatarsal data they are fitted to.

The slope parameter, *k*, is a dimensionless value that describes the profile of the cortical bone. The intercept parameter *m* naturally depended on both *k* and the overall thickness of the cortical bone. The parameters were found using linear least squares. Due to the intrinsic dependence between *k* and *m*, we only considered *k* as a descriptor of the cortical bone profile, which described the longitudinal cortical thickness gradient (CTG). The motivation for using CTG as a descriptor for the progression of arthritis is visualised in Fig. 5, where we see a clear shift in the slope of the cortical thickness in a single arthritic paw as a function of time. To capture the overall thickness of the cortical bone, we used the cortical thickness index (CTI) defined as


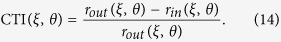


This is an established, dimensionless measure that has been shown to correlate with arthritic progression^20^,^21^. We took the average CTI across the entire bone, both in *ξ* and *θ* direction, to get an overall thickness of the cortical bone. In contrast to the fitted parameter *m*, the average CTI is not necessarily connected with CTG. We can consider cases where CTG changes while the average CTI remains unaltered due to local bone degradation or growth.

We tracked the metatarsals from approximately 3.5 mm above the metatarsophalangeal joint and then went as close to the joint as possible, limited by the scanning angle and the anatomy of the bone. In practice this meant that we had to stop our analysis ~0.75 mm above the joint. From an anatomical point of view, the division of the bone into a cortical piece and bone marrow stops close to the joints, where the spongy bone starts. In the spongy bone there is no division into an inner and an outer perimeter, thus the thickness of the cortical bone is not well-defined.

All image analysis steps, bone orthogonalization as well as calculations of CTG and CTI were done in Python. The code is available from the authors upon request.

## Results

### Measurements of Bone Thickness

In Fig. 5 we plot the evolution of cortical bone thickness, Ψ(*ξ*), for the metatarsals of the right hind paw of animal M29147. It shows that the thickness of the bone close to the proximal phalange decreases as the disease progresses. On the other hand, the thickness higher up on the bone, towards the tarsals, seems to be largely unchanged. This led to the idea that the gradient of the linear function 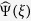, CTG, should be larger for arthritic bones than for non-arthritic ones. If the hypothesis of differing gradients were incorrect, differences in average CTI could be another candidate to describe quantitative differences in cortical bones between arthritic and non-arthritic metatarsals, because a general thinning would have been reflected in this measure. In Table 1, we list the values of CTG and average CTI for the 3rd metatarsal from the right paw of animal M29147 at various time points. The time course of these values is a representative example showing the trend of increasing CTG that was observed qualitatively when the profile of the cortical bone was investigated. In G6PI induced arthritis the inflammation is starting around day 10 post-immunisation^25^ and we noticed a clear increase in CTG at days 18 and 35 compared to earlier time points. The CTI measure did not show a clear trend for the example in Table 1.

### Population statistics

**Figure 6:**
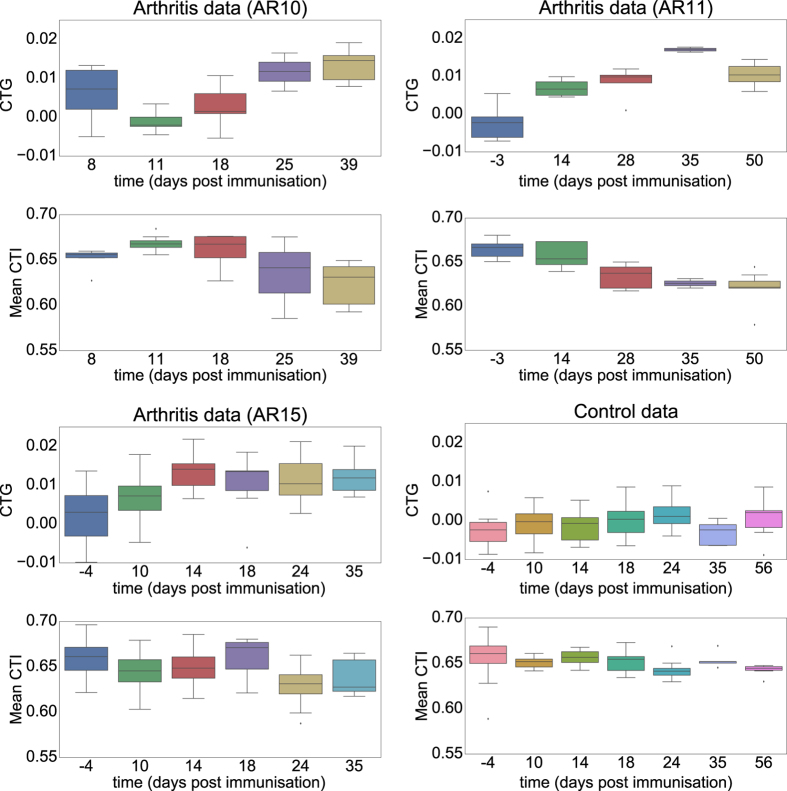
The average values of CTG and CTI for different days post immunisation. The data is split into the different experiments AR10, AR11, AR15 and control.

**Figure 7:**
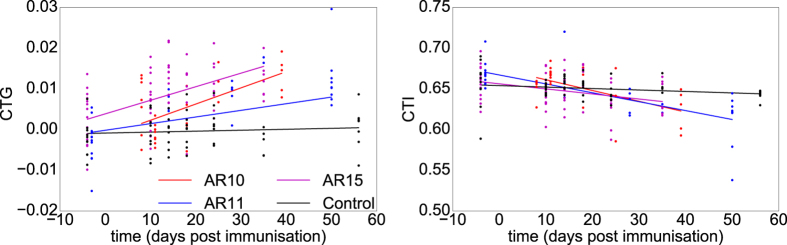
Values and linear fits of CTG and CTI divided across the experiments.

To examine the overall properties of metatarsals, we collected all values of CTG and CTI from the 3rd metatarsal of all paws made on a specific day post-immunisation. The 3rd metatarsal is normally the longest and by picking a single metatarsal we avoided the dependence between data that can be caused by investigating multiple bones from one paw. In Fig. 6, we plot the values for CTG and CTI for various days post-immunisation for the three experiments and the control. The general trend was that CTG indeed increased as the disease progressed while CTI decreased in two of the three arthritis experiments. In the case of AR15 and the control group there was no obvious decrease in the average CTI. For the control group neither CTG nor the average CTI appeared to change over time. We used linear mixed-effect models (LMMs) to quantify CTG and CTI, *i.e*. we considered each paw to be a random variable and looked at the fixed effect of time and whether the animal was immunised or not (control versus arthritic animals) including the interaction term. The interaction term quantifies, whether the change in the measurement was different between conditions, *e.g*. did CTG increase significantly more as a function of time for arthritic paws than for controls. Residual plots did not reveal any obvious deviations from normality or homoscedasticity (see Figs S3 and S4 in the Supplementary Material). For CTG neither time nor immunisation on their own turned out to be significant, *p* > 0.2, while the interaction term between immunisation and time was significantly different between conditions, *p* < 10^−6^. In the scatter plot with linear fits for CTG in Fig. 7 this is illustrated by clear separation between control animals (black line and points) and the immunised animals (AR10 red, AR11 blue and AR15 magenta lines and points) as time progresses. For CTI, the interaction term between immunisation and time was also significant, *p* < 10^−4^, while time and immunisation on their own were not significant factors with *p* > 0.08 for both. The significant difference in interaction between time and immunisation status confirmed the qualitative observations that for arthritic paws CTG increased and average CTI decreased with time while both measures stayed more or less constant in the control group.

The comparison between arthritic paws and controls pointed out a significant difference both for values of CTG and CTI independently. However, in Figs 6 and 7 we see clear differences in CTI between the arthritis experiments. Metatarsals from AR15 had an almost constant average CTI throughout the course of the experiment while AR10 and AR11 had a clear trend of decreasing CTI. To examine the differences between individual experiments, we fitted two LMMs with time and experiment as fixed effects and the individual paws as random variables. We were focusing on the interaction between experiment and time when comparing the experiments, as well as the Bonferroni correction of the *α*-level for the six comparisons that gave significance if *p* < 0.0083. Between the three arthritis experiments there were no significant differences either when considering CTG or CTI (*p* > 0.039). When comparing the individual arthritis experiments with the control experiments, we found that all values were significantly different from the control when looking at CTG (*p* < 10^−4^). For CTI, AR10 and AR11 were significantly different from the control (*p* < 10^−3^) while AR15 was not (*p* ≈ 0.05835). Note that this is far from the Bonferroni corrected *α*-level that requires that *p* < 0.0083. For full details of the *p*-values between individual experiments we refer to Tables S1 and S2 in the Supplementary Material. This confirms the trend we saw in Figs 6 and 7 that CTI did not seem to change very much in the experiment AR15. In contrast to CTI, for CTG AR15 had the highest degree of significant difference between control and arthritis experiment. Between AR15 and control we obtained the p-value *p* ≈ 2.63 · 10^−7^ in the interaction term between time and experiment. For AR10 and AR11 these values were *p* ≈ 2.27 · 10^−6^ and *p* ≈ 3.13 · 10^−5^, respectively.

## Discussion

In this study we described how a combination of *μ*CT and semi-automated image analysis can serve as a tool for longitudinal studies of arthritis in mouse models. The study provided a unique analysis method of longitudinal global and local bone degradation. The key factor for performing the longitudinal analysis was that the animals were not injured during data acquisition. In most cases BMD is used as a measure for degradation but in this study we have demonstrated that, by investigating the profile of the cortical bone from *in vivo μ*CT data, we can quantify the arthritic progression. Here we focused on the cortical bone from metatarsals as the metatarsophalangeal joints are among the areas that are known to be most strongly affected by G6PI induced arthritis^5^. The investigation of such regions is also of interest in RA since the early signs of the disease are most visible in the small joints of the hands and the feet of patients^36^. The texture based segmentation used in this study is fully automated and does not require any manual adjustments. During the metatarsal isolation process, the metatarsals had to be manually identified and the positions of the metatarsophalangeal joints had to be determined. However, compared to the judgements of thresholds or manual removal of artefacts that are required by other studies^2^,^7^,^18^, this is a task that can be performed quickly and does not require any expertise in image analysis and/or mouse anatomy. The orthogonalization of the slices allowed for automatic measurements of the thickness of the cortical bone without any user intervention or knowledge about how the paw was oriented in the scanner. The local orthogonalization only assumed that the bone is approximately straight locally, which is important as the metatarsals are not perfectly straight in their entire length, as can be seen in Fig. 2. The algorithm presented here was tailored to create a pipeline that measures the cortical bone thickness with a minimal amount of parameter adjustments between data sets. To our knowledge, no current software implements the texture based segmentation that we here used to successfully solve the segmentation task.

The orthogonalization of the slices made it possible to automatically measure the cortical profile at given positions along the metatarsal. In this study, we chose to quantify changes in the cortical bone due to G6PI induced arthritis by the longitudinal gradient of the cortical bone thickness (CTG) and by the average cortical thickness index (CTI)^20^. CTG describes the observation that the metatarsals appeared to be more affected by arthritis close to the joints and that most visible lesions occur there^25^. To reduce the dimensionality of the cortical profile, we averaged the thickness around the bone and expressed the thickness as a function of distance to the metatarsophalangeal joint. To capture any general thinning of the bone we also measured the average CTI of the metatarsal. We demonstrated that CTG is more sensitive in separating arthritic metatarsals from non-arthritic metatarsals than CTI. For CTG, all individual experiments showed a significant increase in the parameter compared to control, while CTI for the experiment AR15 was not different from the control. The difference in significance of average CTI between control experiments and the individual arthritis experiments is naturally a reason for concern as all mice were of the same type, housed under identical conditions with similar physical conditions (see Table 2 in the Methods section). The statistical power decreases with the smaller cohorts, which were used to examine significance between individual experiments, thereby increasing the risk for type II errors (false negatives). For all three experiments the animals were manually judged to respond to the immunisation and develop arthritis. Furthermore, between the arthritis experiments neither CTI nor CTG was significantly different, indicating that at least a certain degree of bone damage took place in all experiments. The fact that for CTG the strongest level of significance was found between AR15 and the control animals, indicates that the arthritic damage may have been more localized to the joint regions in AR15 compared to AR10 and AR11. It is known that animal stress levels and response to arthritis can vary because of factors such as gender of the animal handler^37^ or seasonal variations^38^,^39^. In this study, animals from experiments AR10 and AR11 were immunised in winter while experiment AR15 animals were immunised in summer. For experiments AR10 and AR11 the main handler was male while in AR15 and controls the main handlers were female.

We suggest that CTG is a useful measure in an experimental setting where not only arthritic and control animals are examined but also treatment protocols are applied. The fact that CTI was not able to detect a significant difference between the experiment AR15 and control means that experiments with and without treatment may be indistinguishable. CTG can be an additional quantitative measure in such an experimental setting. In this study all animals were scanned at the same CT-machine and therefore any changes in registered cortical bone profile were due to changes in the animals over time. If comparisons are to be made between different laboratories, care must be taken that the cortical profiles are equally measured across scanners^40^,^41^. Here, CTG and CTI calculations, as well as image analysis were all combined in a Python framework, but the calculation of the measurements could also be separated from the image analysis. If commercial software, such as Imaris (Bitplane AG, Zurich, Switzerland) or VGStudio MAX (Volume Graphics GmbH, Heidelberg, Germany) is available, the cortical thickness may be acquired and the CTG could be calculated externally. We are not aware of any open source software that is able to measure the cortical bone thickness using locally orthogonal slices. The ImageJ plugin BoneJ can be extended to include such a functionality^42^, but the current version of this plugin can only align an entire structure along its principal axis, which means that the accuracy of measurement depends on the overall straightness of the structure.

Therefore, our conclusion is that CTG is a measure that should be taken into account when evaluating experimental arthritis. Besides CTI that was considered here, bone roughness^43^, bone volume^16^ or trabecular structure^14^,^18^ are examples of measures that could be considered additionally. It may be overly optimistic to expect that a single measure is enough to evaluate the arthritic progression in animal experiments, but perhaps through the use of machine learning techniques multiple measurements can be effectively evaluated in a combined fashion^44^,^45^.

## Methods

### Experimental design

We used data from three separate arthritis experiments, AR10, AR11 and AR15, and one additional control experiment. All animal studies were approved in accordance with German animal protection laws and were approved by the Federal State Authority of Thuringa and ethics committee (number 02–045/08 for AR10 and AR11, for control and AR15 the number was 02–001/14). Female inbred DBA/1 mice were housed under standard conditions in individually ventilated cages and fed with normal mouse chow and water *ad libitum*. Animals were cared for in accordance with the principles outlined by the European Convention for the protection of vertebrate animals used for experimental purposes. The number of animals used in each experiment, the weight and age of the mice is listed in Table 2. The mice were immunised with G6PI at day zero inducing a poly-arthritic disease^25^. Scans were performed using a small-animal multimodality PET/CT system (Preclinical Solutions, Siemens Healthcare Molecular Imaging, Knoxville, TN, USA). The CT module consists of a cone-beam X-ray *μ*CT source (50-*μ*m focal spot size) and a 3,072 × 2,048-pixel X-ray detector. In our *μ*CT imaging protocol, we used an axial–transaxial resolution of 2,048 × 2,048-pixels, 80 kV at 500 *μ*A, 360° rotation and 360 projections per bed position for paw CT scans. CT images were reconstructed using a Shepp-Logan filter and cone-beam–filtered back projection. For AR10 the animals were scanned at days 8, 11, 18, 25 and 39, the animals scanned at day 8 were only scanned on this day. In experiment AR11 the scanning days were −2, −3, 14, 28, 35 and 50. In AR15 and control, the animals were scanned at days −4, 10, 14, 18, 24 and 35. In the arthritis experiments all animals were immunised at day 0, while six control animals were left un-immunised in order to provide longitudinal controls. During scanning, all animals where kept under anaesthesia by 1.5% isoflurane (Deltaselect, Dreieich, Germany) vaporized in oxygen (1.5 l/min).

### Data

The data set consisted of 175 paw scans from 37 animals, each animal had one or both paws scanned between one and seven times during the course of the experiments. Each *μ*CT-slice was represented by an image, *I(x, y*), that showed the cross-section of the paw at height *z* parallel to the *x*-*y*-plane. Hence, the scan of one paw was set in an original space with base vectors **e**_*x*_, **e**_*y*_ and **e**_*z*_. Each slice was grayscale with *M* × *N* pixels. The exact values of *M* and *N* vary but most images had 300 < *M, N* < 500. In the *z* direction we had between 334 and 602 slices for each paw. The resolution in all directions (*x, y* and *z*) was 14.599 *μ*m.

### Statistics

We used a linear mixed-effect model (LMM)^46^,^47^ to look at the differences between experiments. LMMs are often used in longitudinal studies to examine differences between conditions^46^,^48^,^49^,^50^,^51^,^52^ and allowed us to examine the cortical bone profile even though scans were not always performed at the same days post immunisation. A simple linear model would not have been appropriate as repeated measuring of paws breaks the assumption of independence between data points^53^. As random effects we considered the different individual paws in the study to determine differences between conditions. The *p*-values for the fixed effects were calculated using F-test based on Satterthwaite’s approximation of the degrees of freedom^49^,^54^. The *α*-level indicating significance was set to *p* < 0.05. If multiple comparisons were made, for example when looking at differences between the individual experiments, significance was evaluated using the Bonferroni corrected *α*-level^55^. Statistical analysis was performed in R (R Core Team, 2012) using the lme4 package for the LMM^56^.

## Additional Information

**How to cite this article:** Svensson, C.-M. *et al*. Quantification of arthritic bone degradation by analysis of 3D micro-computed tomography data. *Sci. Rep.* **7**, 44434; doi: 10.1038/srep44434 (2017).

**Publisher's note:** Springer Nature remains neutral with regard to jurisdictional claims in published maps and institutional affiliations.

## Supplementary Material

Supplementary Information

## Figures and Tables

**Table 1:** The CTG and CTI values for the 3rd metatarsal of the right paw from animal M29147 shown in Fig. 5.

Day	−4	10	18	35
CTG	0.006	0.007	0.010	0.014
CTI	0.65	0.63	0.68	0.62

**Table 2:** Details of the animal experiments. All numbers refer to day 0.

Experiment	AR10	AR11	AR15	Control
Number of animals	10	11	11	6
Weight (g)	17.8 ± 0.8	16.6 ± 1.2	17.6 ± 0.5	16.1 ± 0.8
Age (weeks)	9–13	9–13	10–13	12–13
