# Supplementary material for: Quantification of arthritic bone degradation by analysis of 3D micro-computed tomography data
Source: Sci Rep. 2017 Mar 14;7:44434. doi: 10.1038/srep44434 (PMC5349516; doi:10.1038/srep44434)
Supplement: Supplementary Information [file srep44434-s1.pdf]

Quantification of arthritic bone degradation by analysis of 3D micro-computed tomography data: Supplementary material

Carl-Magnus Svensson[1,†], Bianca Hoffman[1,2,3,†], Ingo M. Irmler[4], Maria Straßburger[5], Marc Thilo Figge[1,3,‡,*], and Hans Peter Saluz[1,2,‡]

[1]Applied Systems Biology, Leibniz Institute for Natural Product Research and Infection Biology - Hans-Knöll-Institute, Beutenbergstrasse 11a, 07745 Jena, Germany
[2]Cell and Molecular Biology,Leibniz Institute for Natural Product Research and Infection Biology - Hans-Knöll-Institute, Beutenbergstrasse 11a, 07745 Jena, Germany
[3]Friedrich Schiller University Jena, Germany
[4]Institute of Immunology, University Hospital Jena, Leutragraben 3, 07743 Jena, Germany
[5]Transfer Group Anti-infectives, Leibniz Institute for Natural Product Research and Infection Biology - Hans-Knöll-Institute, Beutenbergstrasse 11a, 07745 Jena, Germany
[*]thilo.figge@leibniz-hki.de
[†]these authors contributed equally to this work
[‡]these authors contributed equally to this work

ABSTRACT

Supplementary material for the article submitted to Nature Scientific Reports. This material presents validation of the use of texture based segmentation, motivates the use of linear mixed models (LMMs) and gives all p-values for the differences between individual experiments.

Texture based segmentation

In Fig. S 1 we show two example μCT slices, their manual segmentation and the segmentations results using the texture based method described in the main manuscript and a threshold based segmentation method. The threshold is automatically determined using Otsu's method[1] and after binarization morphological opening is performed in an attempt to find individual objects. Despite the opening operation, the thresholding tends to merge bones that lie close to each other, an issue that the texture based segmentation does not seem to have. To quantify the difference we manually segmented 10 different μCT slices from five different paws distributed over different experiments, times and animals. For each object found in the manual segmentation we calculate the Dice coefficient to the object in respective automatic segmentation that has the most overlap. The Dice coefficient[2] for two sets of pixels, A and B, is defined as

$$D_c(A,B) = \frac{2|A \cup B|}{|A| + |B|}, \tag{1}$$

where $|\cdot|$ denotes the size of the set. In Fig. S 2 we have plotted the Dice coefficients for both segmentation methods and we can see that the texture based segmentation is significantly better than the threshold version. Significance was measured across 53 objects using a two-tailed Wilcoxon test giving p$\approx$ 0.0047.

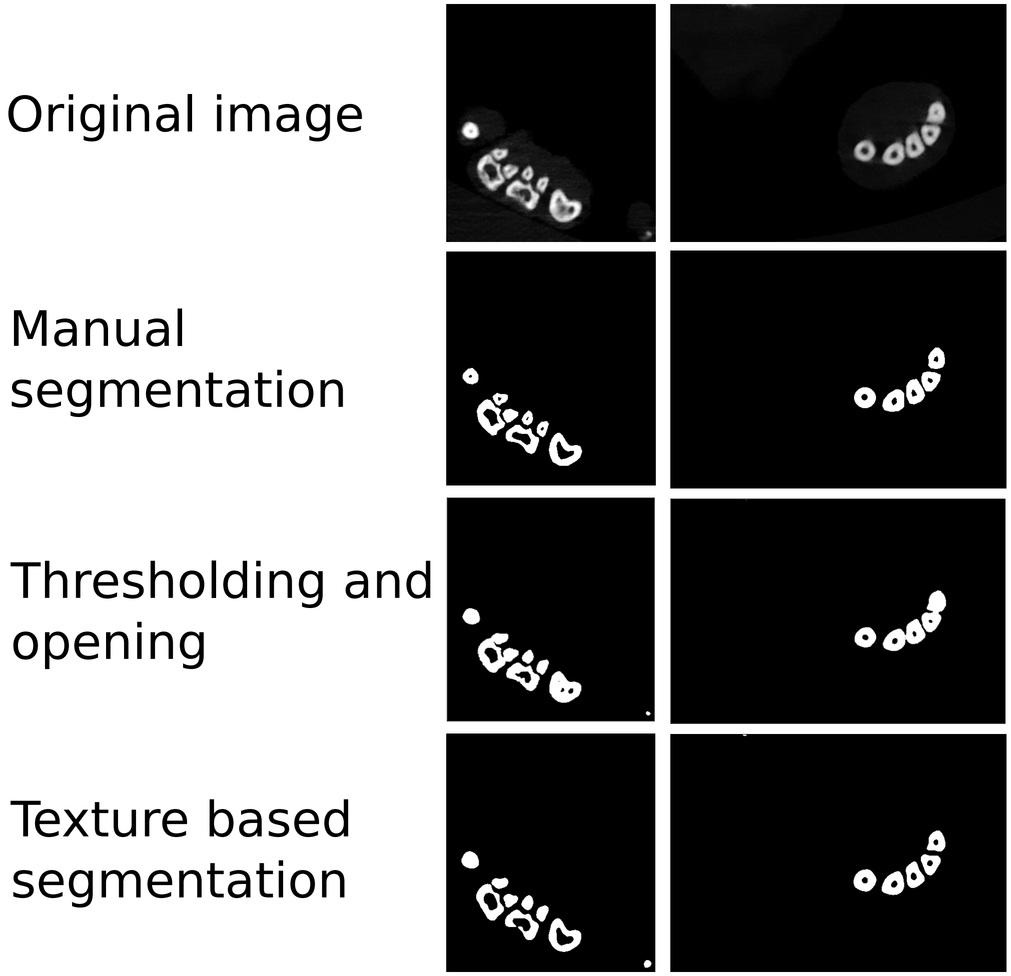

Figure 1. *Examples of segmentation results for two different μCT slices using thresholding and texture based segmentation.*

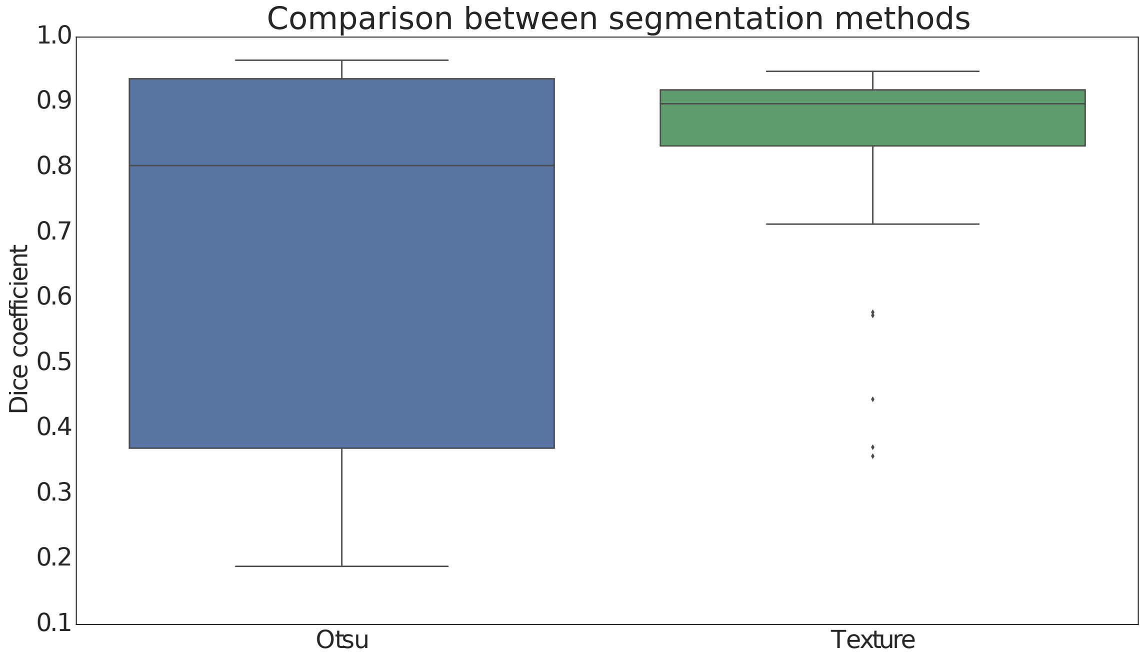

Figure 2. *The Dice coefficient between manually segmented objects and best matching objects in the automatically obtained segmentation with Otsu thresholding and texture based segmentation. Boxes show the limits for the upper and lower quartiles of the distribution and whiskers show the range or 1.5 times the interquartile range. Outliers are shown as diamonds. The texture based segmentation has significantly higher Dice coefficient,* $p \approx 0.0047$ *(Wilcoxon, N=53).*

Data normality and homoscedasticity

To show homoscedasticity we made scale-location plots for our four linear mixed models (LMM). The first LMM is CTI with immunisation status, arthritic animal or not, and time as fixed effects. Then we investigate the difference between individual experiments by using experiment names (AR10, AR11, AR15 and control) as fixed effect together with time. The same fixed effects are naturally used for CTG giving us the four LMMs. In Fig. S 3 we see that neither of the four LMMs show any structure suggesting heteroscedasticity.

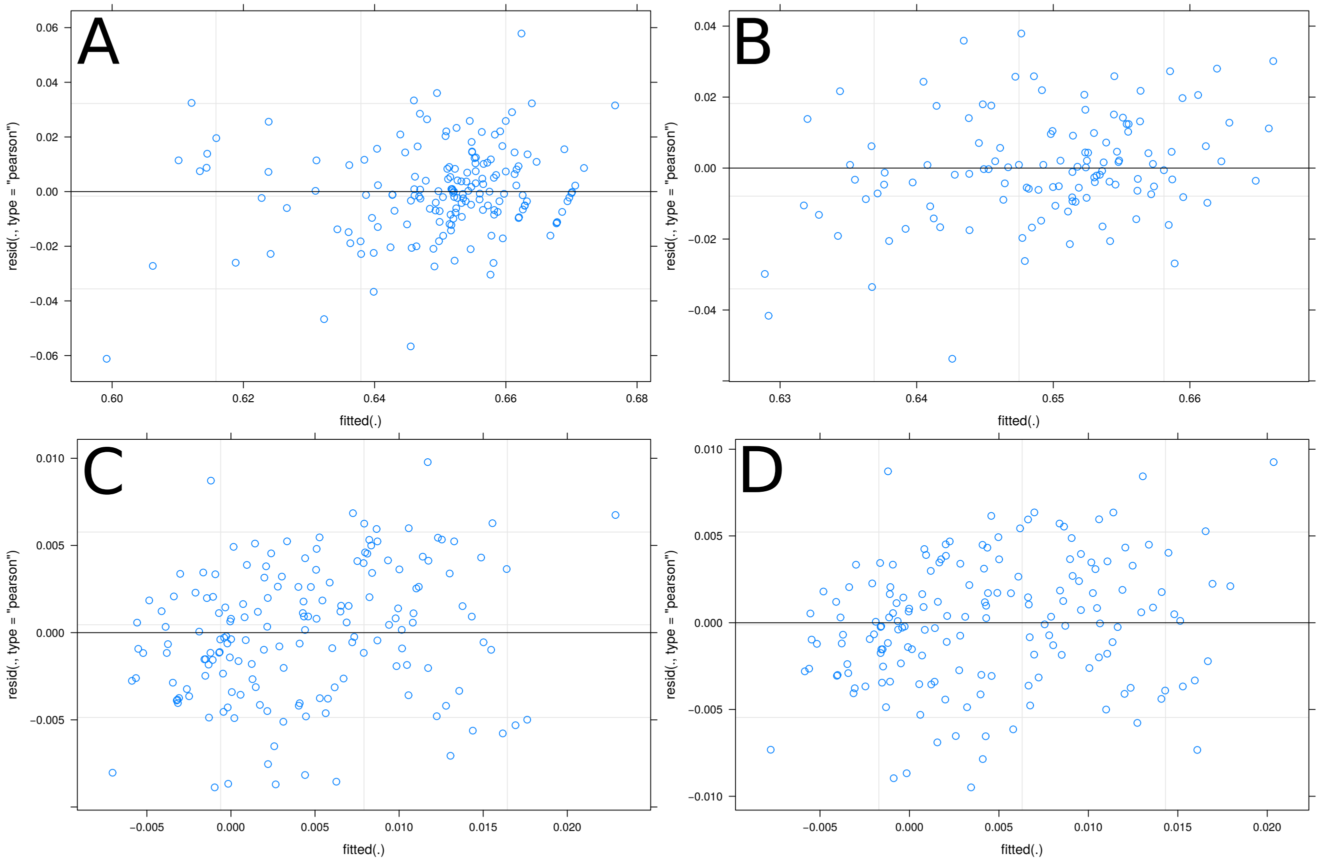

Figure 3. *Scale-location plots for* **A** *CTI with days and immunisation status as fixed effects,* **B** *CTI with days and experiment name as fixed effects,* **C** *CTG with days and immunisation status as fixed effects and* **D** *CTG with days and experiment name as fixed effects*

To check for deviations from normality we made Q-Q plots of the model residuals against theoretical normal quantiles for the four LMMs. In Fig. S 4 we see that the residuals almost exclusively stay within the 95% confidence intervals for normality plotted as dashed lines.

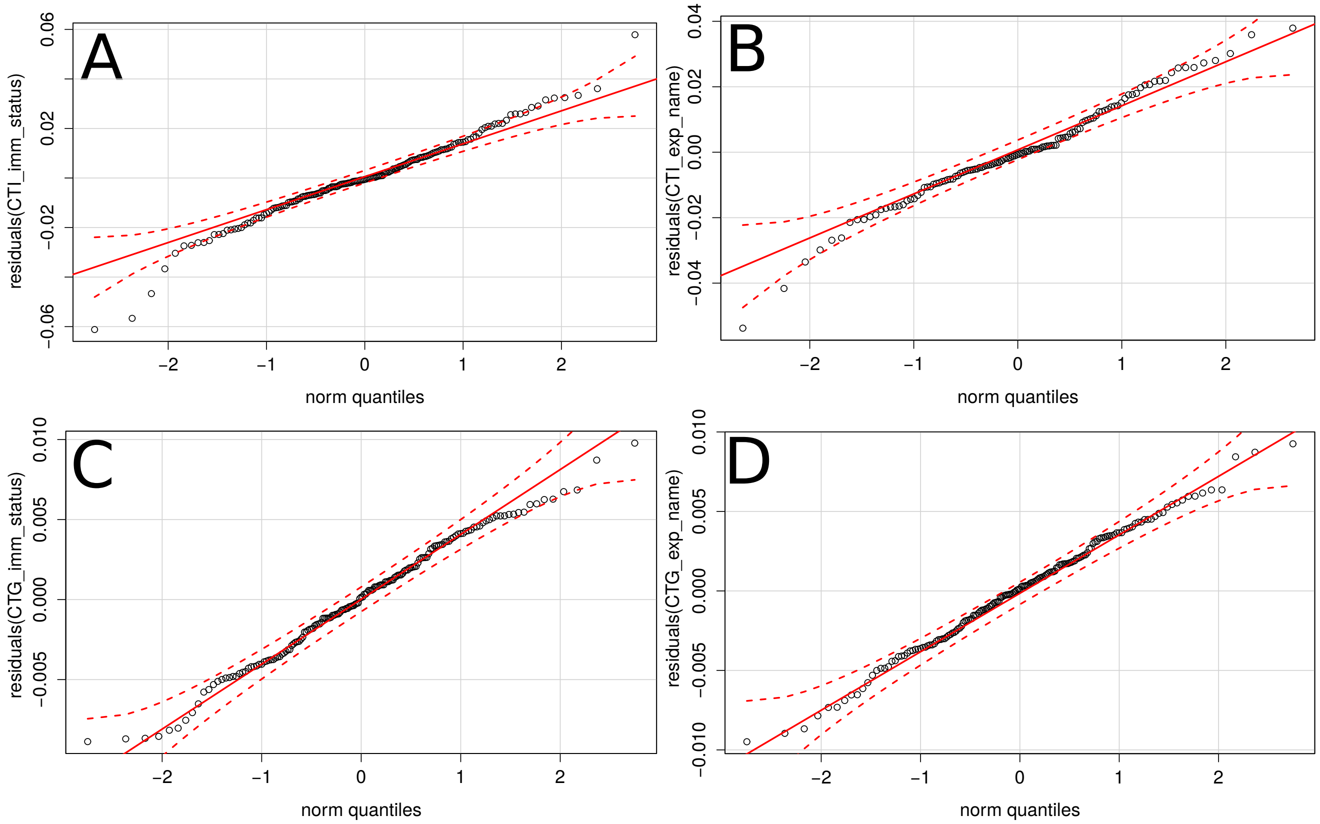

Figure 4. *Q-Q plots for* **A** *CTI with days and immunisation status as fixed effects,* **B** *CTI with days and experiment name as fixed effects,* **C** *CTG with days and immunisation status as fixed effects and* **D** *CTG with days and experiment name as fixed effects. The solid red line show the position of a perfect normal distribution and dashed lines show the 95% confidence intervals for normality.*

Significance between experiments

Here we present the p-values for the interaction term from the LMMs describing differences in CTG and CTI between individual experiments. As we are making in total six comparisons we Bonferroni correct the α-level giving us significance if $p < 0.0083$. As stated in the main manuscript CTG has significant differences between all individual experiments and control while for CTI the difference between AR15 and control is non-significant. There are no significant differences between arthritis experiments either in CTI or CTG.

Table S 1. The p-values between individual experiments of the LMM interaction term for CTG. Bonferroni corrected α-level requires $p < 0.0083$ for significance.

	AR10	AR11	AR15	Control
AR10	-	0.039	0.22	$2.27 \cdot 10^{-6}$
AR11		-	0.24	$3.13 \cdot 10^{-5}$
AR15			-	$2.63 \cdot 10^{-7}$

Table S 2. The p-values between individual experiments of the LMM interaction term for CTI. Bonferroni corrected α-level requires $p < 0.0083$ for significance.

	AR10	AR11	AR15	Control
AR10	-	0.44	0.043	$6.34 \cdot 10^{-4}$
AR11		-	0.055	$1.71 \cdot 10^{-5}$
AR15			-	0.058

References

1. Otsu, N. A threshold selection method from gray-level histograms. *IEEE T. Sys. Man Cybern.* **9**, 62–66 (1979).
2. Dice, L. R. Measures of the amount of ecologic association between species. *Ecology* **26**, 297–302 (1945).
